# Identification and verification of hub genes associated with the progression of non-small cell lung cancer by integrated analysis

**DOI:** 10.3389/fphar.2022.997842

**Published:** 2022-09-13

**Authors:** Xie Mengyan, Ding Kun, Jing Xinming, Wei Yutian, Shu Yongqian

**Affiliations:** ^1^ Department of Oncology, The First Affiliated Hospital of Nanjing Medical University, Nanjing, China; ^2^ Department of Molecular Cell Biology and Toxicology, Center for Global Health, School of Public Health, Nanjing Medical University, Nanjing, China; ^3^ Key Laboratory of Modern Toxicology of Ministry of Education, School of Public Health, Nanjing Medical University, Nanjing, China; ^4^ Jiangsu Key Lab of Cancer Biomarkers, Prevention and Treatment, Collaborative Innovation Center for Cancer Personalized Medicine, Nanjing Medical University, Nanjing, China; ^5^ Department of Neurosurgery, The First Affiliated Hospital of Nanjing Medical University, Nanjing, Jiangsu, China

**Keywords:** NSCLC, hub genes, bioinformatics, integrated analysis, biomarker

## Abstract

**Objectives:** Lung cancer is one of the most common cancers worldwide and it is the leading cause of cancer-related mortality. Despite the treatment of patients with non-small cell lung carcinoma (NSCLC) have improved, the molecular mechanisms of NSCLC are still to be further explored.

**Materials and Methods:** Microarray datasets from the Gene Expression Omnibus (GEO) database were selected to identify the candidate genes associated with tumorigenesis and progression of non-small cell lung carcinoma. The differentially expressed genes (DEGs) were identified by GEO2R. Protein-protein interaction network (PPI) were used to screen out hub genes. The expression levels of hub genes were verified by GEPIA, Oncomine and The Human Protein Atlas (HPA) databases. Survival analysis and receiver operating characteristic (ROC) curve analysis were performed to value the importance of hub genes in NSCLC diagnosis and prognosis. ENCODE and cBioPortal were used to explore the upstream regulatory mechanisms of hub genes. Analysis on CancerSEA Tool, CCK8 assay and colony formation assay revealed the functions of hub genes in NSCLC.

**Results:** A total of 426 DEGs were identified, including 93 up-regulated genes and 333 down-regulated genes. And nine hub genes (CDC6, KIAA0101, CDC20, BUB1B, CCNA2, NCAPG, KIF11, BUB1 and CDK1) were found to increase with the tumorigenesis, progression and cisplatin resistance of NSCLC, especially EGFR- or KRAS-mutation driven NSCLC. Hub genes were valuable biomarkers for NSCLC, and the overexpression of hub genes led to poor survival of NSCLC patients. Function analysis showed that hub genes played roles in cell cycle and proliferation, and knockdown of hub genes significantly inhibited A549 and SPCA1 cell growth. Further exploration demonstrated that copy number alterations (CNAs) and transcription activation may account for the up-regulation of hub genes.

**Conclusion:** Hub genes identified in this study provided better understanding of molecular mechanisms within tumorigenesis and progression of NSCLC, and provided potential targets for NSCLC treatment as well.

## 1 Introduction

Malignant tumors are important diseases that seriously threaten human health, among which, lung cancer occupies an important position. According to the 2020 CA reports, the global morbidity and mortality of lung cancer ranked second and first, respectively, with 2.21 million new cases and 1.80 million deaths worldwide (Sung et al., 2021). NSCLC is a subtype of lung cancer based on histopathology, including large cell carcinoma, squamous cell carcinoma (LUSC) and adenocarcinoma (LUAD). NSCLC accounts for 85% of lung cancers and the 5-year survival rate is less than 15% (Duma et al., 2019). In the past 2 decades, the investigation into NSCLC tumorigenesis has made great progress, increasing our understanding of NSCLC treatment strategy. Nowadays, the use of small molecular inhibitors and immunotherapy has brought unprecedented survival benefits to NSCLC patients (Herbst et al., 2018). However, because of individual differences and tumor heterogeneity, the problems of drug toxicities, side effects, single target and drug tolerance are still prominent, and the overall cure rates and survival rates of NSCLC are still low (Konieczkowski et al., 2018).

Overactivated oncogenes and mutated or inactivated tumor suppressor genes account for tumorigenesis. A variety of genes and signal regulatory networks are involved in tumorigenesis, leading to the occurrence and development of NSCLC (Hanahan and Weinberg, 2011). Currently, an increasing number of driver genomic alterations with potential targeted treatments have been identified in NSCLC. For example, activating gene mutations of epidermal growth factor receptor (EGFR), anaplastic lymphoma kinase (ALK), pro-oncogene receptor tyrosine kinase (ROS1) and serine/threonine protein kinase (BRAF) or fusion has now become the target of NSCLC kinase inhibitor therapy (Rotow and Bivona, 2017). However, EGFR mutations were observed in 10–20% of white patients (Dearden et al., 2013), translocations of ALK were identified in 2–7% of patients with NSCLC (Mesaros et al., 2014) and ROS1 in 1–2% of patients with NSCLC (Bergethon et al., 2012). Therefore, it is particularly important to further explore the molecular mechanism of NSCLC tumorigenesis and progression, thus identifying new biomarkers and therapeutic targets.

**FIGURE 1:**
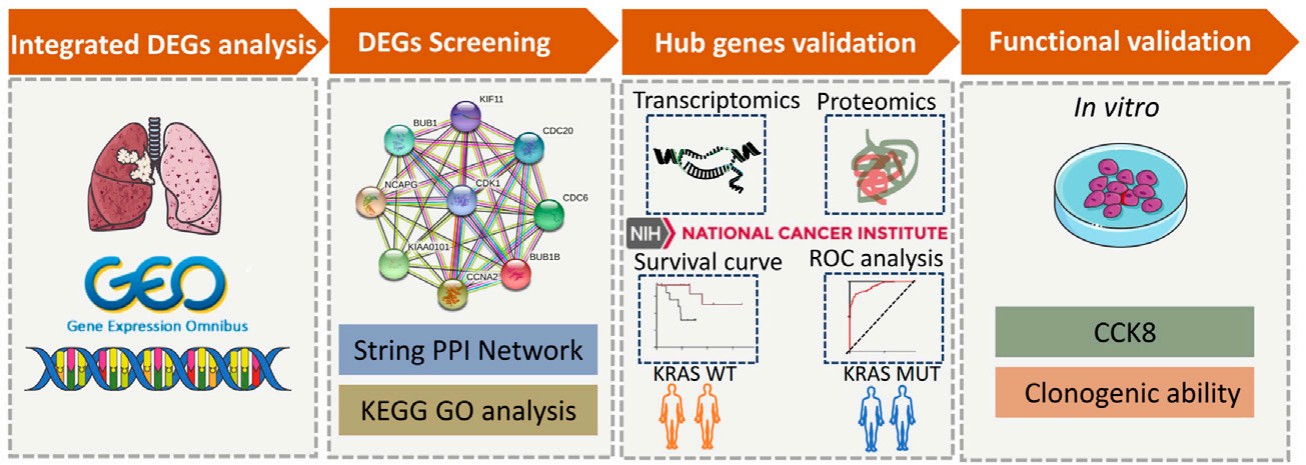
A schematic representation of the research methods. Diagram showing the four main modules in this study, including DEGs analysis, DEGs screening, hub genes validation and functional validation.

In this study, we reanalyzed three gene expression profiles from the GEO repository. The DEGs between NSCLC tissues and normal tissues were confirmed by the GEO2R. We also performed the GO and KEGG pathway analysis to detect the functions of DEGs in NSCLC. Through the PPI network, we found there were nine hub genes (CDC6, KIAA0101, CDC20, BUB1B, CCNA2, NCAPG, KIF11, BUB1, CDK1) in strong connection within the network. Then, the mRNA and protein expression levels in NSCLC were confirm by GEPIA, Oncomine and HPA databases. The level of protein expression was the most important as proteins were genetic products that eventually do their job. HPA collected reliable data on the protein expression of CDC6, CDC20, KIF11, CCNA2, NCAPG, CDK1. Some of these proteins showed strong positive expression in immunohistochemistry of lung cancer, making them promising markers for immunohistochemical diagnosis of NSCLC. Moreover, GEO data regarding gene-edited mouse models revealed that the expression of hub genes increased with the progression of NSCLC, and cohort study in lung cancer showed that the hub genes may be involved in EGFR- or KRAS-mutation driven NSCLC progression. The survival analysis demonstrated that the overexpression of hub genes was positively correlated with worse survival of NSCLC patients. We also validated their significance as biomarkers in NSCLC and confirmed their combined diagnostic value by ROC curve analysis. CNAs, a hallmark in the cancer genome, together with histone modifications and transcription factors, accounted for the ectopic expression of hub genes. The CancerSEA Tool indicated that the hub genes played important roles in cell cycle and cell proliferation, so we designed siRNAs targeting these hub genes to test their functions in NSCLC cell lines. CCK8 assay and colony formation assay both proved that silencing hub genes significantly inhibited A549 and SPCA1 cell growth, demonstrating the promoting abilities of hub genes in NSCLC development. The schematic representation of this study is shown in Figure 1.

## 2 Materials and methods

### 2.1 Microarray data

The GEO database (http://www.ncbi.nlm.nih.gov/geo/) is an international public repository that records and publishes microarrays, next-generation sequencing and other forms of high-throughput functional genomics data (Barrett et al., 2013). We selected the microarray datasets GSE19804, GSE43458 and GSE18842 to screen out candidate DEGs. GSE84447, GSE52248, GSE33479, GSE31210 and GSE161584 datasets were used to verify the expression of hub genes. Detail information of GEO profiles are shown in Supplementary Table S1.

### 2.2 Identification of DEGs

GEO2R is an interactive web tool that allows users to compare two or more sample groups in the GEO series to identify genes that are differentially expressed under different experimental conditions, the results are presented in the form of gene table sorted by significance. Consequently, DEGs between NSCLC tissues and normal tissues were confirmed by the GEO2R. The adjusted *p* values were utilized to reduce the false positive rate using the Benjamin and Hochberg false discovery rate method by default (Wei et al., 2019). We defined the cutoff value as |Log_2_FC (fold change) | ≥ 2 and *p* < 0.05 for further analysis. Then, Venn diagrams showing the number of overlapping DEGs in the three GEO datasets was performed by Venn package (http://bioinformatics.psb.ugent.be/webtools/Venn/). Finally, forest plots and heatmap were performed by the software GraphPad Prim 8.

### 2.3 GO and KEGG pathway of DEGs

The Gene Ontology (GO) is a widely used ontology in the field of bioinformatics, covering three aspects of biology: biological process (BP), cellular component (CC) and molecular function (MF) (Gene Ontology, 2015). Kyoto Encyclopedia of Genes and Genomes (KEGG) is a utility database resource for understanding advanced functions and biological systems (such as cells, organisms, and ecosystems) from the molecular level information, especially large molecular datasets generated by genome sequencing and other high-throughput experimental techniques (Kanehisa et al., 2019). Database for Annotation, Visualization and Integrated Discovery (DAVID, http://David.ncifcrf.gov/) is a bioinformatics resources that contains a large amount of integrated information and analysis tools designed to provide interpretation for large gene and protein lists (Huang Da et al., 2009). GO and KEGG pathway analyses of these DEGs were performed with DAVID.

### 2.4 Protein-protein interaction (PPI) network

To clarify the node molecules that play a key role in the regulation of tumorigenesis and development, we constructed a PPI network. Search Tool for the Retrieval of Interacting Genes (STRING) was used to build PPI networks (Szklarczyk et al., 2011). First, we use STRING to build a PPI network, then use Cytoscape software to visualize the results obtained from the STRING database, and finally use MCODE plugin of Cytoscape software to screen hub genes which have the highest node scores and the strongest connectivity.

### 2.5 Overall survival (OS) of DEGs

The Kaplan Meier plotter (https://kmplot.com/analysis/) can analyze the effect of 54,000 genes on survival in 21 cancer types. Gene expression data and OS data were obtained from GEO and TCGA(Gyorffy et al., 2013). To analyze the prognostic value of hub genes, the tumor and normal tissues were divided into two groups based on median expression (high vs. low expression) and the curves of OS were performed by the Kaplan Meier plotter with the hazard ratio (HR) with 95% confidence intervals (CIs) and log rank *p* value.

### 2.6 Receiver operating characteristic (ROC) curve

The ROC curve is a comprehensive index reflecting the continuous variables of sensitivity and specificity. Area under the ROC curve (AUC) reflects diagnostic value of the test. In general, an AUC above 0.9 is considered a highly accurate diagnostic test. The ROC curve was conducted by Graph Pad Prism 7.0 based on the GEO chip data. The ROC curve of multi-factors was conducted by SPSS statistics 20.0 analyzed by logistic regression.

### 2.7 Cell culture and siRNA transfection

The A549 cell line was cultured in RPMI 1640 medium (Gibco, Carlsbad, CA, United States) and the SPCA1 cell line was cultured in Dulbecco’s modified eagle medium. Cells were supplemented with 100 μg/ml streptomycin, 100 U/ml penicillin and 10% fetal bovine serum (FBS) at 37°C in a humidified atmosphere of 5% CO2. The siRNAs (Supplementary Table S2) targeting hub genes were designed and synthesized by RiboBio (Guangzhou, China). The siRNAs were transfected into the cells with Lipofectamine 3,000 (Invitrogen).

### 2.8 Cell counting kit-8 (CCK8) assay

After 48 h of transfection, A549 and SPCA1 cells (3 × 10^3^) were seeded into 96-well plates (Corning). Then, 10 μL of CCK8 (Beyotime, Jiangsu, China) solution was added to each well at the point of 48 h. After 1 h of incubation at 37°C, the absorbance at 450 nm was measured using an automatic microplate reader (Synergy4; BioTek, Winooski, VT, United States).

### 2.9 Colony formation assay

After 48 h of transfection, A549 (300) and SPCA1 (400) cells were seeded into 6-well plates (Corning). Cells were cultured until the colony reached 2 mm wide. The cells were fixed with 1% paraformaldehyde (Beyotime) for 1 h and stained with crystal violet (Beyotime) for 12 h.

### 2.10 Western blot assay

Proteins in cells were extracted with RIPA lysis buffer (Thermo Fisher). Western blot assays were performed according to details previously reported (Zhou et al., 2008). The immunocomplexes were detected with ECL Western Blotting Substrate (Thermo Fisher), visualized with BIO-RAD (BIO-RAD Gel Doc XR+, United States). The following antibodies were used (1:1000): anti-α-tubulin (Beyotime, AF0001); anti-BUB1 (Proteintech, 13330-1-AP); anti-BUB1B (Proteintech, 11504-2-AP); anti-CCNA2 (Proteintech, 18202-1-AP); anti-CDC6 (Proteintech, 11640-1-AP); anti-CDC20 (Proteintech, 10252-1-AP); anti-CDK1 (Proteintech, 19532-1-AP); anti-KIF11 (Proteintech, 23333-1-AP); anti-KIAA0101 (Santa Cruz, sc-390515); anti-NCAPG (Proteintech, 24563-1-AP).

### 2.11 RNA preparation and quantitative real-time PCR (qRT-PCR)

Total RNA was extracted from the cells using the TRIzol reagent (Invitrogen, MA, United States). Isolated RNA was used for the reverse transcription reaction with HiScript Q RT SuperMix for qPCR (Vazyme, Jiangsu, China). Quantitative RT-PCR was carried out with SYBR Green PCR Master Mix (Vazyme) using an ABI Prism 7,900 Sequence detection system (Applied Biosystems, Canada). GAPDH was used as an internal control, and the results for each sample were normalized to GAPDH expression. The primers are listed in Supplementary Table S3.

## 3 Results

### 3.1 Identification of DEGs and hub genes in NSCLC

#### 3.1.1 DEGs were identified *via* GEO profiles of NSCLC

**FIGURE 2:**
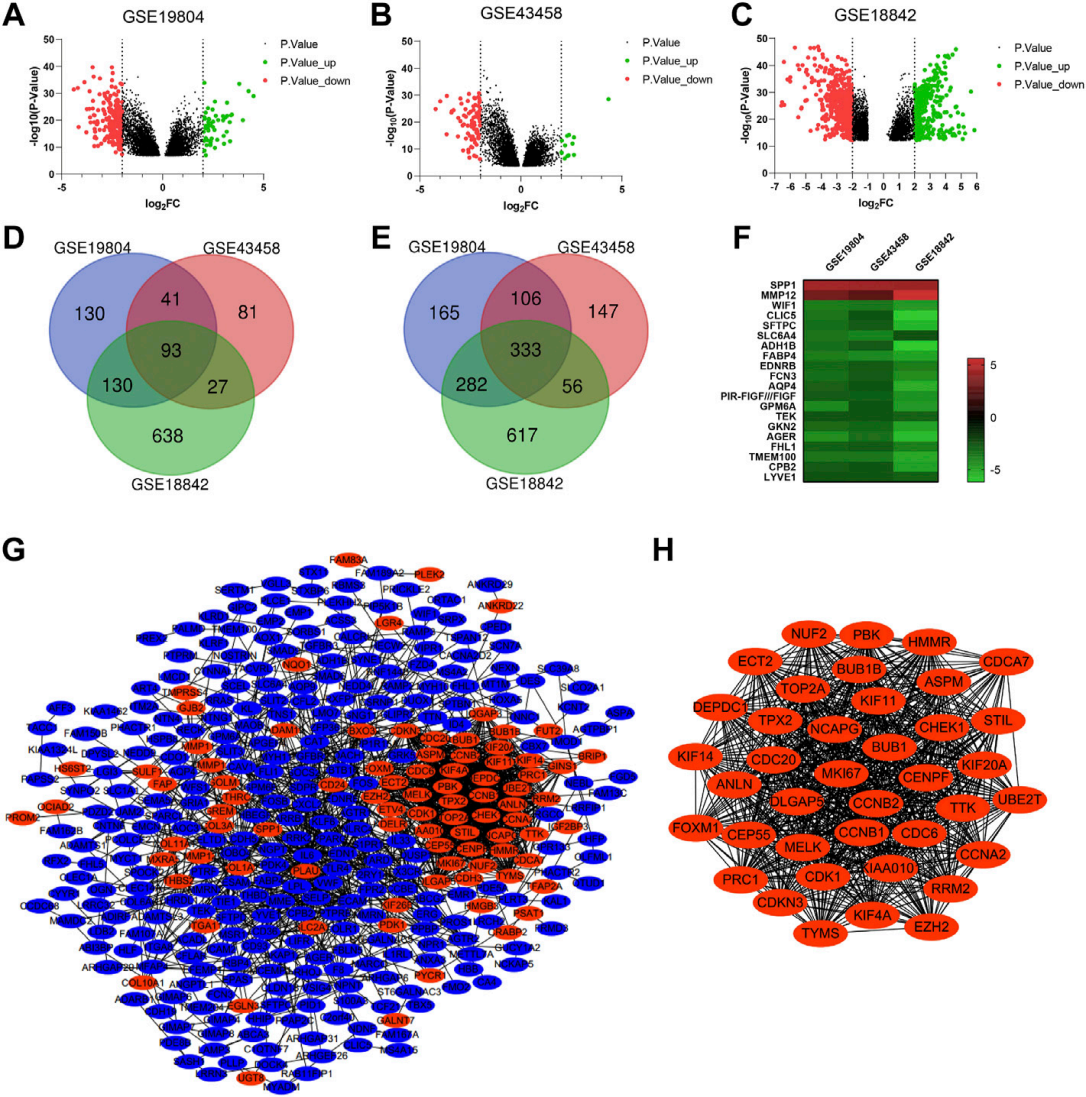
Identification of overlapping DEGs and PPI construction of DEGs. **(A-C)** Volcano plots showing DEGs in NSCLC tissues compared to normal tissues in GSE19804/GSE43458/GSE18842 (|Log_2_FC| ≥ 2 and *p* < 0.05). **(D and E)** Venn diagrams showing the number of overlapping DEGs in the three GEO datasets (D. up-regulated genes E. down-regulated genes). **(F)** Heatmap of the top representative DEGs (|Log_2_FC| ≥ 2.5 and *p* < 0.05). **(G)** PPI network of DEGs constructed by the String and Cytoscape software. **(H)** The most significant module of PPI network based on the score of each node. (Red represents up-regulated, blue represents down-regulated).

Three microarray profiles (GSE19804/GSE43458/GSE18842) were obtained from GEO database. The cut-off was |Log_2_FC| ≥ 2 and *p* value <0.05. A total of 1280, 884 and 2,176 DEGs was observed in GSE19804, GSE43458 and GSE18842, respectively (Figures 2A–C). The Venn diagrams displayed that there were 426 genes found in all three profiles, including 93 up-regulated genes and 333 down-regulated genes in NSCLC tissues compared to normal tissues (Figures 2C, D). Twenty genes were visualized in the heatmap when the cut-off was limited to |Log_2_FC| ≥ 2.5 (*p* < 0.05) (Figures 2F).

For a deeper understanding on the selected DEGs, we performed the GO function and KEGG pathway enrichment analysis (Supplementary Figure S1). Biological process enrichment showed that up-regulated DEGs were mainly enriched in collagen fibril organization, microtubule-based movement and regulation of cell proliferation (Supplementary Figure S1A), while down-regulated DEGs were mainly enriched in transforming growth factor beta receptor signaling pathway, receptor internalization and positive regulation of angiogenesis (Supplementary Figure S1E). By KEGG pathway analysis of DEGs, we found that most of the up-regulated DEGs were enriched in cell cycle and ECM-receptor interaction (Supplementary Figure S1D), while most of the down-regulated DEGs were enriched in cell adhesion molecules and PARP signaling pathway (Supplementary Figure S1F).

#### 3.1.2 Hub genes were identified *via* PPI network and module analysis

To investigate the protein interactions between the screened DEGs, PPI network was constructed by the String and Cytoscape software (Figure 2G). The PPI network of DEGs consisted of 369 nodes and 1835 edges. We used the MCODE plugin to find the most significant module of the PPI network, which includes 39 nodes and 720 edges (Figure 2H). Interestingly, compared to normal tissues, the genes in the most connected module were all up-regulated in NSCLC tissues. We considered the top nine genes as the hub genes based on the score of each node: CDC6 (cell division cycle 6), NCAPG (non-SMC condensin I complex subunit G), KIF11 (kinesin family member 11), KIAA0101, CDC20 (cell division cycle 20), BUB1 (mitotic checkpoint serine/threonine kinase), CDK1 (cyclin dependent kinase 1), CCNA2 (cyclin A2), BUB1B (BUBI mitotic checkpoint serine/threonine kinase B). Go analysis of the top nine genes indicated that these hub genes were mainly associated with cell division and kinase binding (Supplementary Table S4). KEGG pathway enrichment showed that these hub genes were enriched in cell cycle, progesterone-mediated oocyte maturation, oocyte meiosis and viral carcinogenesis (Supplementary Table S4).

### 3.2 Verification of hub genes expression in NSCLC

#### 3.2.1 mRNA and protein expression levels of hub genes increased in NSCLC

**FIGURE 3:**
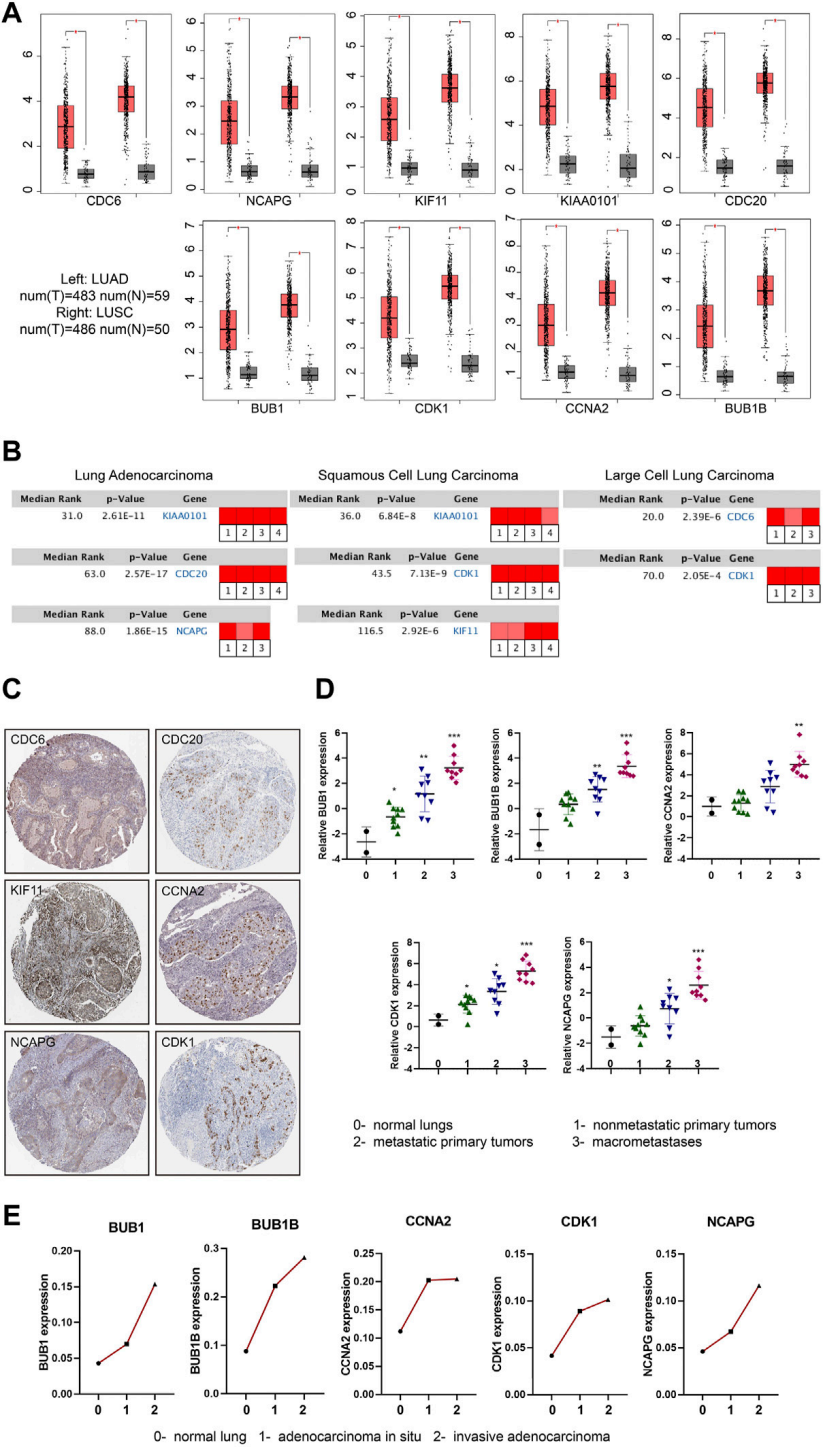
mRNA and protein expression changes of hub genes in NSCLC. **(A)** Boxplots (Red box: tumor tissue; Grey box: normal tissue) showing the expression levels of hub genes based on the TCGA database. The red and grey boxes represent NSCLC and normal tissues, respectively. |Log_2_FC| cutoff = 1, *p* value cutoff = 0.05. **(B)** Rank of hub genes changes in subtypes of NSCLC according to Oncomine database (Red represents up-regulated). |Log_2_FC| cutoff = 1, *p* value cutoff = 0.001. **(C)** Immunohistochemical staining of hub genes in NSCLC tumor cells included in HPA database. **(D)** The expression of hub genes with the progression of LUAD in GSE84447 dataset: 0- normal lungs (*n* = 2), one- nonmetastatic primary tumors (*n* = 10), two- metastatic primary tumors (*n* = 9),3- macrometastases (*n* = 9). **(E)** The expression of hub genes with the progression of LUAD in GSE52248 dataset. Each point represents the average value of the group (each group: *n* = 6). *p < 0.05, **p < 0.01, ***p < 0.001, ****p < 0.0001.

To further confirm the expression level of hub genes in the NSCLC tissues and normal tissues, we took advantage of the online database GEPIA (Tang et al., 2017) (Gene Expression Profiling Interactive Analysis) (http://gepia.cancer-pku.cn/) and Oncomine (http://www.oncomine.org/). We found that the expression levels of the hub genes (CDC6, KIAA0101, CDC20, BUB1B, CCNA2, NCAPG, KIF11, BUB1 and CDK1) were significantly up-regulated in NSCLC tissues compared to normal tissues (Figure 3A). Interestingly, we found the expression levels of hub genes were slightly higher in LUSC than that in LUAD. Based on Oncomine database, we found that all the hub genes were related to NSCLC and were expressed differently in varied pathological types of NSCLC. The most obvious changed genes in LUAD, LUSC and large cell lung carcinoma were KIAA0101, KIAA0101 and CDC6, respectively (Figure 3B; Supplementary Table S5). Besides the expression of mRNAs, we further analyzed the protein expression levels of the hub genes. HPA (http://www.proteinatlas.org/) includes the immunohistochemical information of CDC6, CDC20, KIF11, CCNA2, NCAPG and CDK1 proteins. According to the database, most of the hub genes were undetected or low expression in lung tissues. However, in NSCLC tissues, the positive rates of staining were over 60% (positive cases/total cases: CDC6 6/10, CDC20 11/11, KIF11 10/10, CCNA2 10/11, NCAPG 9/10, CDK1 10/12) (Figure 3C; Supplementary Table S6).

#### 3.2.2 Hub genes expression increased with tumorigenesis, progression and chemotherapy resistance of NSCLC

**FIGURE 4:**
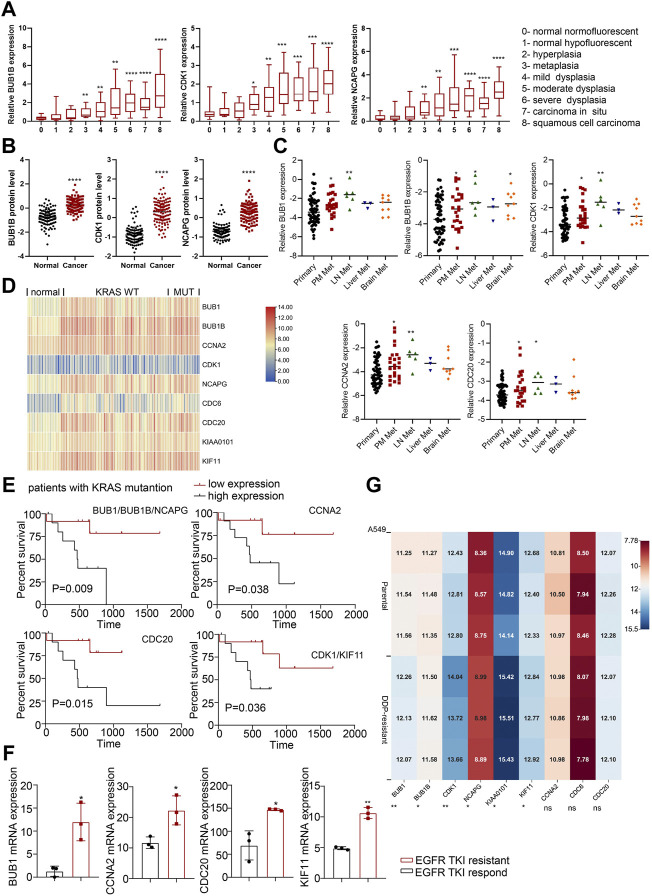
The roles of hub genes in LUSC and in EGFR- or KRAS-mutation driven NSCLC. **(A)** The expression levels of hub genes in different stages of LUSC tumorigenesis (122 samples from 77 patients). **(B)** The protein expression levels of hub genes in LUSC in CPTAC database. **(C)** The expression levels of hub genes (BUB1, BUB1B, CDK1, CCNA2 and CDC20) in lung adenocarcinomas identified patients with dismal prognosis. GSE13213: Primary lung cancer: *n* = 58; PM metastasis: *n* = 24; LN metastasis: *n* = 6; Liver metastasis: *n* = 3; Brain metastasis: *n* = 9. **(D)** Heatmap of hub gene expression in KRAS-mutant and KRAS-wild type lung cancer compared to normal lung tissues (GSE31210). Normal lung: *n* = 20, KRAS MUT: n = 20, KRAS WT: n = 68. **(E)** Kaplan-Meier analysis of hub genes in LUAD with KRAS mutation from TCGA. **(F)** Hub gene expression in EGFR-TKI-sensitive and TKI-resistant lung cancer tissues in GSE161584 dataset. **(G)** Heatmap of hub gene expression in DDP-resistant A549 cells and control cells (GSE108214). *p < 0.05, **p < 0.01, ***p < 0.001, ****p < 0.0001.

We then used the GEO data regarding gene-edited mouse models (GSE84447 and GSE52248) to observe the role of hub gene expression in the occurrence and development of NSCLC. We found the expression of BUB1, BUB1B, CCNA2, CDK1 and NCAPG increased with the continuous progression of LUAD in mice (Figures 3D, E; Supplementary Figures S2A, B). Similarly, the progression data of LUSC (GSE33479) showed that these nine hub genes were highly expressed in LUSC compared with the initial stage of tumorigenesis (Figure 4A; Supplementary Figure S3A). In addition, the protein levels of hub genes (The National Cancer Institute’s Clinical Proteomic Tumor Analysis Consortium, CPTAC) were also high in LUSC (Figure 4B; Supplementary Figure S3B). Considering the high metastasis rate of NSCLC, we further analyzed the relationship between hub genes and distant metastasis of lung cancer (GSE13213). Compared to primary lung cancer, all the expression of hub genes increased in lymph node metastasis samples, and the expression of BUB1, BUB1B, CDK1, CCNA2 and CDC20 were related to peritoneum metastasis (Figure 4C; Supplementary Figure S3C).

KRAS mutations and EGFR mutations are the main mutations driving the development of lung cancer. We analyzed lung cancer cohorts with KRAS mutations (GSE31210) and found that higher hub gene levels (BUB1, BUB1B, NCAPG, CDC20, CDK1, KIF11) were significantly associated with worse OS in lung cancer patients with KRAS mutations (Figures 4D, E; Supplementary Figure S3D). In addition, the expression level of main hub genes (BUB1, CCNA2, CDC20, KIF11) was significantly higher in patients with EGFR TKI-resistant lung cancer tissues (GSE161584) than EGFR TKI-sensitive lung cancer tissues (Figure 4F; Supplementary Figure S3E). Taken together, these hub genes may be involved in EGFR- or KRAS-mutation driven NSCLC progression.

Cisplatin (DDP) resistance is an important reason hindering the chemotherapy efficacy of lung cancer. We analyzed the transcriptome data of A549 cells before and after DDP resistance (GSE108214) to study the expression of hub genes in them. The results showed that BUB1, BUB1B, CDK1, NCAPG, KIAA0101 and KIF11 were highly expressed in DDP-resistant A549 cells (Figure 4G), which indicated that hub genes might promote chemoresistance in NSCLC.

### 3.3 Survival analysis and ROC curve analysis of hub genes in NSCLC

#### 3.3.1 Up-regulated hub genes led to poor OS of NSCLC patients

**FIGURE 5:**
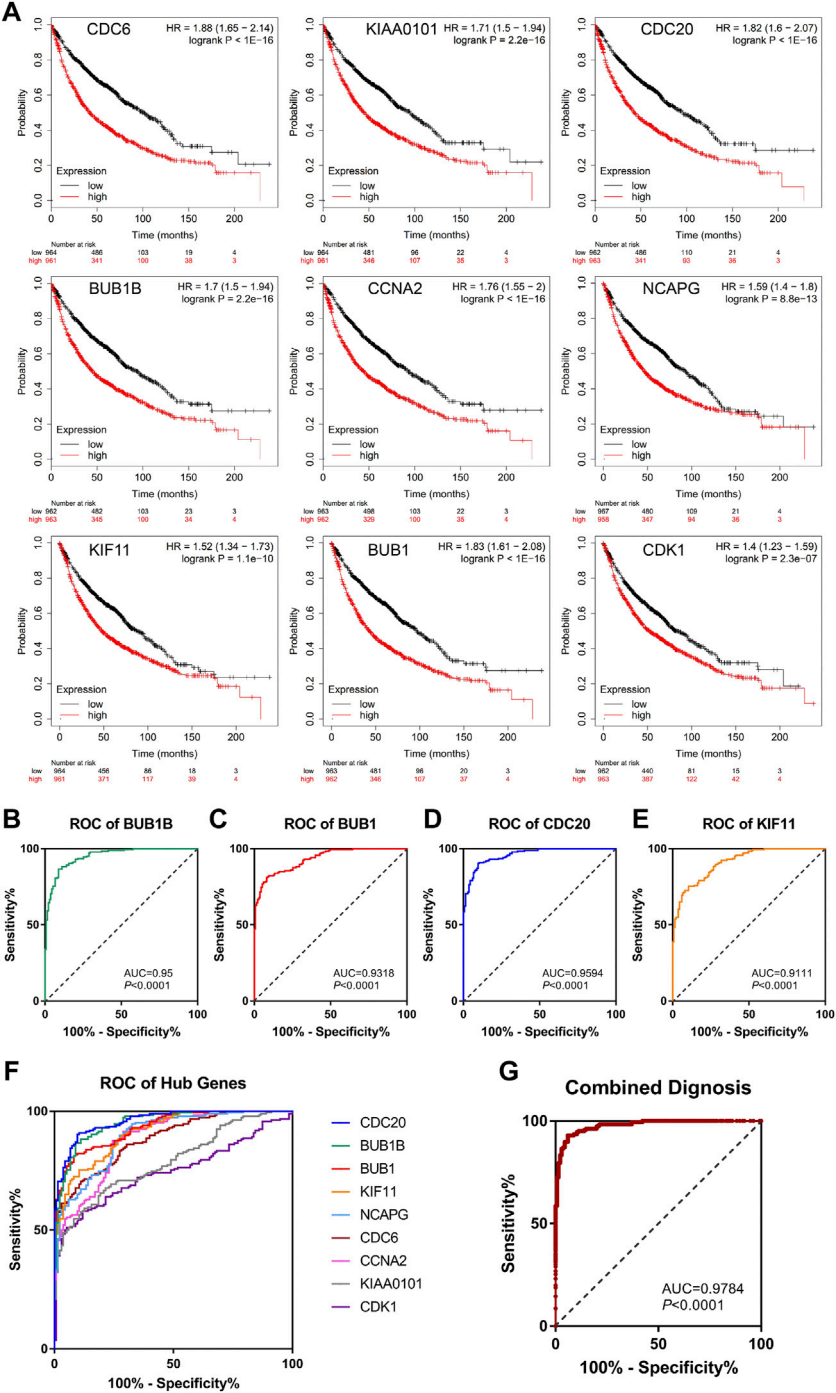
Survival analysis and ROC curve analysis of hub genes in NSCLC. **(A)** Kaplan-Meier survival curves of OS based on the hub genes (CDC6, KIAA0101, CDC20, BUB1B, CCNA2, NCAPG, KIF11, BUB1, CDK1) expression using the online bioinformatics tool Kaplan-Meier Plotter. **(B–F)** Individual ROC curve of hub genes according to the chip data of GSE19804, GSE43458 and GSE18842. **(G)** Combined ROC curve of hub genes according to the chip data of GSE19804, GSE43458 and GSE18842.

To further confirm the correlation with the survival of clinical patients, we used the online bioinformatics tool Kaplan-Meier Plotter (http://kmplot.com/). The results showed that NSCLC patients with higher expression levels of CDC6 [HR = 1.88 (1.65–2.14), *p* < 1e-16], KIAA0101 [HR = 1.71 (1.5–1.94), *p* = 2.2e-16], CDC20 [HR = 1.82 (1.6–2.07), *p* < 1e-16], BUB1B [HR = 1.7 (1.5–1.94), *p* = 2.2e-16], CCNA2 [HR = 1.76 (1.55–2), *p* < 1e-16], NCAPG [HR = 1.59 (1.4–1.8), *p* = 8.8e-13], KIF11 [HR = 1.52 (1.34–1.73), *p* = 1.1e-10], BUB1 [HR = 1.83 (1.61–2.08), *p* < 1e-16], CDK1 [HR = 1.4 (1.23–1.59), *p* = 2.3e-07] had worse OS (Figure 5A).

#### 3.3.2 Hub genes were valuable indicators for NSCLC

Next, we used GEO chip data (GSE19804, GSE43458 and GSE18842) to determine the feasibility of hub genes as biomarkers for NSCLC. ROC curve of independent factors showed that all nine hub genes had moderate or above diagnostic value for NSCLC (Figures 5B–F), among which CDC20, BUB1B, BUB1 and KIF11 had significant diagnostic value, and the areas under the curve were 0.9594 [95% CI: 0.9413 to 0.9775], 0.95 [95% CI: 0.9284 to 0.9716], 0.9318 [95% CI: 0.9069 to 0.9567] and 0.9111 [95% CI: 0.8816 to 0.9406], respectively (*p* < 0.0001). However, considering that the diagnosis of diseases should not rely on a single indicator, but often requires the joint diagnosis of multiple indicators, we incorporated nine hub genes into the NSCLC diagnosis system, fitted a regression equation (z = 0.031* BUB1B+ 2.666* CDC20 + 0.138* KIF11 + 0.879* BUB1+ 2.224* CDC6- 1.922* CDK1- 0.793* CCNA2+ 2.052* KIAA0101–0.983* NCAPG- 26.192) for the diagnosis score of NSCLC through logistic regression analysis, and conducted ROC analysis on the prediction probability of the regression analysis (Figure 5G). We found that the area under the curve increased to 0.9784 (95% CI: 0.966 to 0.9908, *p* < 0.0001). Compared with ROC analysis of a single indicator, multi-factor combined diagnosis has higher accuracy and application value.

### 3.4 Investigation into upstream regulatory mechanisms of hub genes

#### 3.4.1 Copy number alterations

**FIGURE 6:**
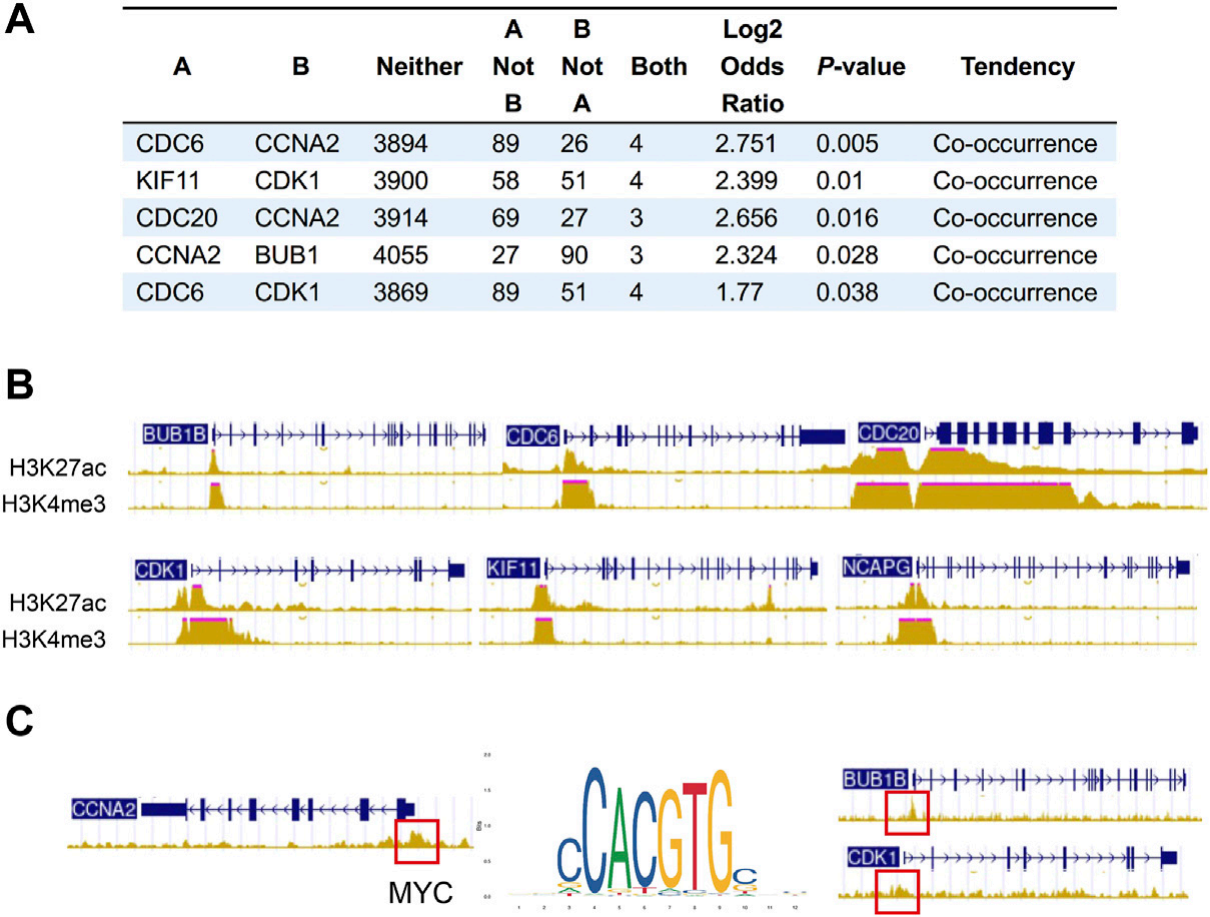
Co-occurrence of hub gene alterations in NSCLC and ChIP-seq of histone and transcription factors in A549 cells. **(A)** Five pairs of hub genes tending to have concurrent CNAs in NSCLC. **(B)** The characteristic peaks (fold change over control) of H3K4me3 and H3K27ac at the promoter region of BUB1B, CDC6, CDC20, CDK1, NCAPG and KIF11. **(C)** The characteristic peaks (fold change over control) of MYC at the promoter region of BUB1B, CDK1 and CCNA2.

We next sought to address the correlation between CNA frequencies and expression levels of the hub genes via online cancer genomics data sets cBioPortal (http://www.cbioportal.org/). We found that there are different degrees of Hub genes changes in the genomes of 6,322 patients from 18 NSCLC studies. Among them, the gene alterations of hub genes were less than 2%, and the CNA types of each gene were different. While CNA events in deep depletion and amplification rarely or less occurred, most hub genes often underwent shallow depletion or copy gain (Supplementary Figures S4A–D). In addition, we also found that the alterations of CDC6 and CCNA2, KIF11 and CDK1, CDC20 and CCNA2, CCNA2 and BUB1, and CDC6 and CDK1 had the tendency of co-occurrence (Figure 6A).

#### 3.4.2 Transcriptional activation

Besides CNA, chromatin immunoprecipitation (ChIP) assay of A549 (ENCSR778NQS, ENCSR000DPD) from ENCODE database verified high expression of H3K4me3 and H3K27ac at the promoter region of BUB1B, CDC6, CDC20, CDK1, NCAPG and KIF11, which may lead to the upregulation of these hub genes (Figure 6B). Some overexpressed transcription factors in lung cancer also activates the expression of hub genes. For example, characteristic peak of MYC was demonstrated by TF ChIP-seq in A549 (ENCSR000DYC) at the promoter region of BUB1B, CDK1 and CCNA2 (Figure 6C). So, transcriptional activation may partially account for the upregulation of hub genes.

### 3.5 The biological functions of hub genes in NSCLC

#### 3.5.1 Hub genes were involved in cell cycle and cell proliferation

**FIGURE 7:**
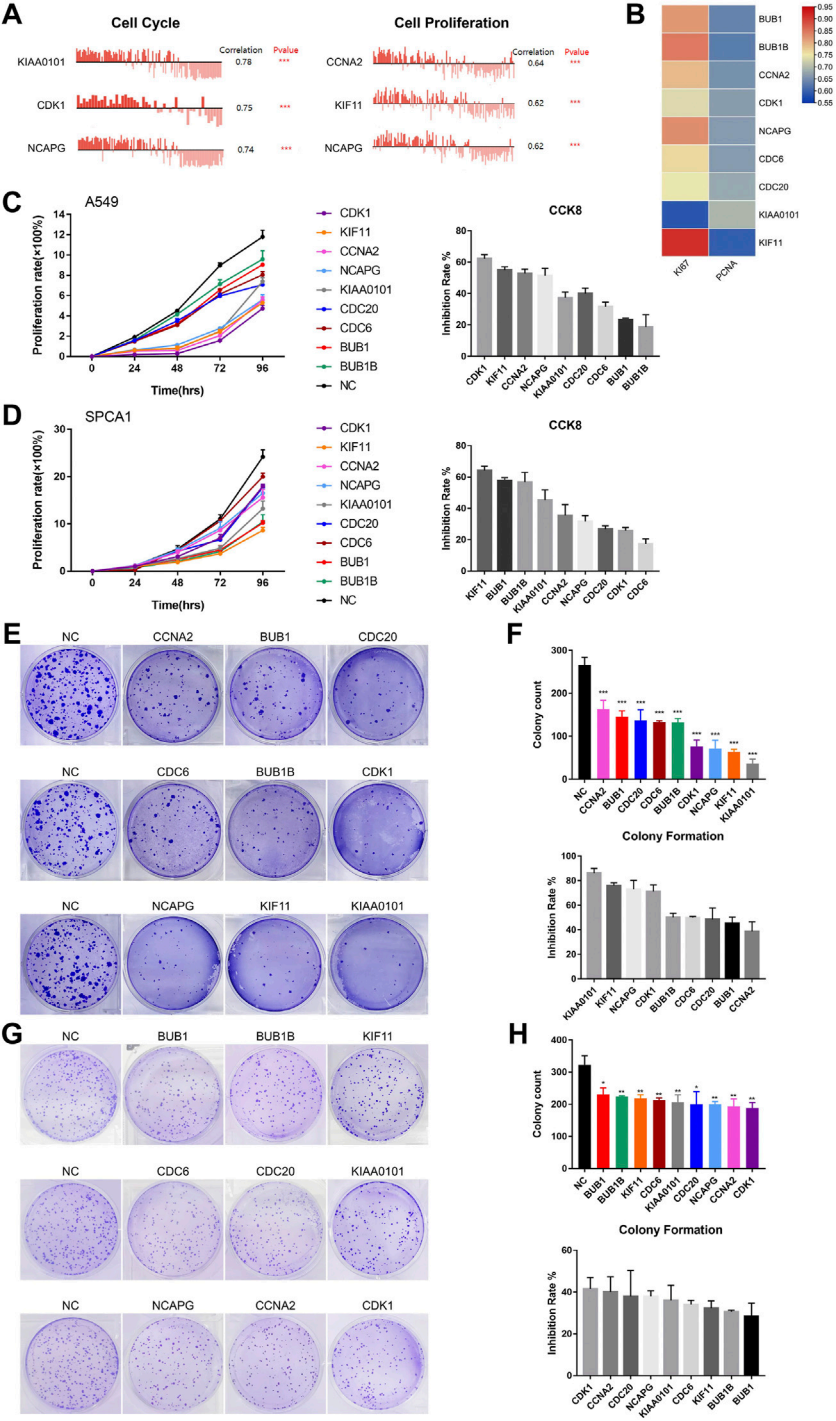
The biological functions of hub genes in NSCLC. **(A)** The correlation between cell cycle, cell proliferation and hub gene expression at single cell level in NSCLC via CancerSEA database. **(B)** Correlation between Ki-67, PCNA and hub gene expression based on TCGA-LUAD analysis, *n* = 526. Statistical significance was tested using Pearson’s correlation coefficient. Correlation scores were analyzed by the Pearson correlation test. **(C)** Assessment of the proliferation of A549 cells transfected with siRNAs#1 targeting hub genes by CCK8 assay (Left). The inhibition rate of each siRNA#1 on cell proliferation in CCK8 assay (Right). **(D)** Assessment of the proliferation of SPCA1 cells transfected with siRNAs#1 targeting hub genes by CCK8 assay (Left). The inhibition rate of each siRNA#1 on cell proliferation in CCK8 assay (Right). **(E,F)** Assessment of the colony formation ability of A549 cells transfected with siRNAs#1 targeting hub genes by colony formation assay. The inhibition rate of each siRNA#1 on colony formation. **(G,H)** Assessment of the colony formation ability of SPCA1 cells transfected with siRNAs#1 targeting hub genes by colony formation assay. The inhibition rate of each siRNA#1 on colony formation. *p < 0.05, **p < 0.01, ***p < 0.001, ****p < 0.0001.

In order to detect the function of hub genes in NSCLC, we first used the CancerSEA Tool. The results showed that the single cell expression levels of hub genes were positively correlated with the progression of cell cycle, proliferation, invasion, DNA damage and EMT (Supplementary Figure S4E). Cell cycle and cell proliferation were the most relevant functions, among which KIAA0101 and CCNA2 took the first place, respectively (Figure 7A). Expression levels of hub genes were also positively correlated with the Ki-67 and PCNA expression (proliferation markers), which was in agreement with the opinion that hub genes were key factors in lung cancer cell proliferation (Figure 7B).

#### 3.5.2 Down-regulated hub genes inhibited NSCLC cell growth

Then, we constructed two si-RNAs (si-RNA#1 and si-RNA#2) of each hub gene and transfected them into A549 and SPCA1 cells. The efficiency of si-RNAs were verified by qRT-PCR and western blot (Supplementary Figure S5). CCK8 assay revealed that silencing hub genes significantly inhibited the proliferation rate of NSCLC cells (Figures 7C, D; Supplementary Figures S6A, B). The inhibiting effect of each si-RNAs was different in 2 cell lines, but we found that the proliferation-promoting function of KIF11 was one of the most obvious in both cell lines, which was in consistent with the results in CancerSEA. We also conducted colony formation assay and the result showed that silencing hub genes remarkably decreased the numbers of colony (Figures 7E−H; Supplementary Figures S6C–F). CCK8 and colony formation assays demonstrated that the hub genes played important roles in NSCLC cell growth.

## 4 Discussion

Despite advances in targeted therapy and immunotherapy in advanced NSCLC patients, early detection and prevention can significantly improve clinical outcomes and bring economic benefits to patients. However, there are many challenges in early-stage NSCLC. About 20% early-stage lung cancer patients faced recurrence and metastasis after surgery worldwide (Hirsch et al., 2017; Herbst et al., 2018). Therefore, more comprehensive understanding of lung cancer based on transcriptomics can fill the gap between genomic abnormalities and oncogenic protein mechanisms. In this study, we screened a series of hub genes based on GEO datasets and these hub genes are associated with prognosis of NSCLC. Further pathway analysis revealed that most of the up-regulated DEGs were enriched in cell cycle and ECM-receptor interaction, which are vital events in hallmarks of tumor. In addition, combination of these genes can better predict the prognosis of NSCLC.

CDC20, CDK1, CCNA2 and CDC6 are four cyclin-related genes in screened hub genes. At the heart of the cell cycle, CDKs drive cells through different phases of the cell cycle. Importantly, CDK1 can replace other CDKs and has been found to be sufficient to drive the mammalian cell cycle (Santamaria et al., 2007; Haneke et al., 2020). CDC6 is a key replication licensing factor that plays a vital role in regulating DNA replication (Borlado and Mendez, 2008). Moreover, E6AP-low/CDC6-high/P16ink4a-low protein abundance profiles are associated with hypomethylation of the gene encoding P16ink4a (CDKN2A) and poorer prognosis in NSCLC patients (Lim and Townsend, 2020). Recent studies have shown that CDC20 promotes several types of cancer progression, including glioma and lung cancer (Wang et al., 2013; Wang et al., 2015). Aurora B phosphorylates BUB1 to facilitate spindle assembly checkpoint signaling, and target Aurora B kinase can prevent and overcome resistance to EGFR inhibitors in lung cancer (Tanaka et al., 2021; Roy et al., 2022). KIF11, known as mitotic spindle-specific protein, and its oral inhibitor 4SC-205 demonstrates anti-tumor activity and enhances targeted therapy in primary and metastatic neuroblastoma models (Masanas et al., 2021). Dysregulation of NCAPG may contribute to the progression of hepatocellular carcinoma and gastric cancer (Gong et al., 2019; Wu et al., 2021). KIAA0101 interacts with UBCH10 to regulate non-small cell lung cancer proliferation by disrupting spindle assembly checkpoint function (Lei et al., 2020). These results suggest potential therapeutic benefits of these candidates in NSCLC.

EGFR inhibitors are important targeted drugs for the treatment of lung cancer, which can significantly improve the prognosis of patients. However, the use of EGFR inhibitors will inevitably lead to mutation resistance and toxic side effects (Ercan et al., 2015; Liu et al., 2018). Acquired resistance to EGFR TKIs occurs through the selection of pre-existing resistant clones and the evolution of resistance persistence (DTP) that survives treatment through adaptive mechanisms (Hata et al., 2016). Over time, DTP can acquire resistance through mutational or non-mutational mechanisms. It is particularly vital to find drug resistance targets to improve the sensitivity of inhibitors. In this study, we found that the expression of BUB1, CCNA2, CDC20 and KIF11 increased after inhibitor resistance compared with inhibitor-sensitive lung cancer samples, and these targets were associate with cell cycle, cell proliferation, DNA damage and EMT. EMT is characterized by histological changes in a subset of EGFR-mutant NSCLC patients with acquired resistance to EGFR inhibitors, either independently or in combination with genetic resistance mechanisms such as EGFR T790M(Sequist et al., 2011; Tulchinsky et al., 2019). Therefore, targeting these gene expression may alleviate EGFR-TKI resistance to a certain extent.

Lung cancer is a molecular heterogeneous disease, extensive heterogeneity in the development hinders drug development and affects the prognosis of patients (Diaz et al., 2012; Mcgranahan et al., 2015). Given the tumor heterogeneity, targeting single molecular does not cure lung cancer patients. Therefore, multiple hub genes were screened in our study. Various studies have reported cases of multi-target sequential intervention in the treatment of various cancer types: for patients with triple-negative breast cancer expressing MYCN, the combination of BET inhibitors and MEK inhibitors can synergistically inhibit tumor growth (Schafer et al., 2020); in patients with melanoma, combined inhibition of PD-1, BRAF and MEK can significantly prolong patient survival (Dummer et al., 2020); for advanced lung cancer, combined intervention of TRA, ROS1 and ALK targets reduces brain metastasis in patients (Drilon et al., 2017). Multi-factor combined diagnosis in our screened hub genes had higher accuracy and application value, all hub genes were associated with cell proliferation and cell cycle. Knocking down these hub genes suppressed tumor growth based on colony formation and CCK8 assays. Therefore, combining these hub genes may have better predictive value for patients with NSCLC, and targeting hub genes for combination research are needed.

Our study also has some limitations that need to be noted. First, there is a lack of validation of the lung cancer cohort, as it was analyzed only by public data. Second, suitable inhibitors can be screened for intervention based on the expression of targeted genes in the future, which can be used for translational medicine research. In addition, our studies need further functional biological validations.

In conclusion, our study screened out nine hub genes associated with NSCLC progression, which make a contribution to the molecular subtyping of NSCLC. It also provides new biomarkers for the prognosis and effective targets for the treatment of NSCLC.

## Data Availability

The datasets presented in this study can be found in online repositories. The names of the repository/repositories and accession number(s) can be found in the article/Supplementary Material.

## References

[B1] Barrett T. Wilhite S. E. Ledoux P. Evangelista C. Kim I. F. Tomashevsky M. (2013). NCBI GEO: Archive for functional genomics data sets--update. Nucleic Acids Res. 41, D991–D995. 10.1093/nar/gks1193 23193258 PMC3531084

[B2] Bergethon K. Shaw A. T. Ou S. H. Katayama R. Lovly C. M. Mcdonald N. T. (2012). ROS1 rearrangements define a unique molecular class of lung cancers. J. Clin. Oncol. 30, 863–870. 10.1200/JCO.2011.35.6345 22215748 PMC3295572

[B3] Borlado L. R. Mendez J. (2008). CDC6: From DNA replication to cell cycle checkpoints and oncogenesis. Carcinogenesis 29, 237–243. 10.1093/carcin/bgm268 18048387

[B4] Dearden S. Stevens J. Wu Y. L. Blowers D. (2013). Mutation incidence and coincidence in non small-cell lung cancer: meta-analyses by ethnicity and histology (mutMap). Ann. Oncol. 24, 2371–2376. 10.1093/annonc/mdt205 23723294 PMC3755331

[B5] Diaz L. A. Jr. Williams R. T. Wu J. Kinde I. Hecht J. R. Berlin J. (2012). The molecular evolution of acquired resistance to targeted EGFR blockade in colorectal cancers. Nature 486, 537–540. 10.1038/nature11219 22722843 PMC3436069

[B6] Drilon A. Siena S. Ou S. I. Patel M. Ahn M. J. Lee J. (2017). Safety and antitumor activity of the multitargeted pan-TRK, ROS1, and ALK inhibitor entrectinib: Combined results from two phase I trials (ALKA-372-001 and STARTRK-1). Cancer Discov. 7, 400–409. 10.1158/2159-8290.CD-16-1237 28183697 PMC5380583

[B7] Duma N. Santana-Davila R. Molina J. R. (2019). Non-small cell lung cancer: Epidemiology, screening, diagnosis, and treatment. Mayo Clin. Proc. 94, 1623–1640. 10.1016/j.mayocp.2019.01.013 31378236

[B8] Dummer R. Lebbe C. Atkinson V. Mandala M. Nathan P. D. Arance A. (2020). Combined PD-1, BRAF and MEK inhibition in advanced BRAF-mutant melanoma: Safety run-in and biomarker cohorts of COMBI-i. Nat. Med. 26, 1557–1563. 10.1038/s41591-020-1082-2 33020648

[B9] Ercan D. Choi H. G. Yun C. H. Capelletti M. Xie T. Eck M. J. (2015). EGFR mutations and resistance to irreversible pyrimidine-based EGFR inhibitors. Clin. Cancer Res. 21, 3913–3923. 10.1158/1078-0432.CCR-14-2789 25948633 PMC4791951

[B10] Gene Ontology C. (2015). Gene ontology Consortium: Going forward. Nucleic Acids Res. 43, D1049–D1056. 10.1093/nar/gku1179 25428369 PMC4383973

[B11] Gong C. Ai J. Fan Y. Gao J. Liu W. Feng Q. (2019). NCAPG promotes the proliferation of hepatocellular carcinoma through PI3K/AKT signaling. Onco. Targets. Ther. 12, 8537–8552. 10.2147/OTT.S217916 31802891 PMC6801502

[B12] Gyorffy B. Surowiak P. Budczies J. Lanczky A. (2013). Online survival analysis software to assess the prognostic value of biomarkers using transcriptomic data in non-small-cell lung cancer. PLoS One 8, e82241. 10.1371/journal.pone.0082241 24367507 PMC3867325

[B13] Hanahan D. Weinberg R. A. (2011). Hallmarks of cancer: The next generation. Cell 144, 646–674. 10.1016/j.cell.2011.02.013 21376230

[B14] Haneke K. Schott J. Lindner D. Hollensen A. K. Damgaard C. K. Mongis C. (2020). CDK1 couples proliferation with protein synthesis. J. Cell Biol. 219, e201906147. 10.1083/jcb.201906147 32040547 PMC7054999

[B15] Hata A. N. Niederst M. J. Archibald H. L. Gomez-Caraballo M. Siddiqui F. M. Mulvey H. E. (2016). Tumor cells can follow distinct evolutionary paths to become resistant to epidermal growth factor receptor inhibition. Nat. Med. 22, 262–269. 10.1038/nm.4040 26828195 PMC4900892

[B16] Herbst R. S. Morgensztern D. Boshoff C. (2018). The biology and management of non-small cell lung cancer. Nature 553, 446–454. 10.1038/nature25183 29364287

[B17] Hirsch F. R. Scagliotti G. V. Mulshine J. L. Kwon R. Curran W. J. Jr. Wu Y. L. (2017). Lung cancer: Current therapies and new targeted treatments. Lancet 389, 299–311. 10.1016/S0140-6736(16)30958-8 27574741

[B18] Huang Da W. Sherman B. T. Lempicki R. A. (2009). Systematic and integrative analysis of large gene lists using DAVID bioinformatics resources. Nat. Protoc. 4, 44–57. 10.1038/nprot.2008.211 19131956

[B19] Kanehisa M. Sato Y. Furumichi M. Morishima K. Tanabe M. (2019). New approach for understanding genome variations in KEGG. Nucleic Acids Res. 47, D590–D595. 10.1093/nar/gky962 30321428 PMC6324070

[B20] Konieczkowski D. J. Johannessen C. M. Garraway L. A. (2018). A convergence-based framework for cancer drug resistance. Cancer Cell 33, 801–815. 10.1016/j.ccell.2018.03.025 29763622 PMC5957297

[B21] Lei H. Wang K. Jiang T. Lu J. Dong X. Wang F. (2020). KIAA0101 and UbcH10 interact to regulate non-small cell lung cancer cell proliferation by disrupting the function of the spindle assembly checkpoint. BMC Cancer 20, 957. 10.1186/s12885-020-07463-3 33008389 PMC7532574

[B22] Lim N. Townsend P. A. (2020). Cdc6 as a novel target in cancer: Oncogenic potential, senescence and subcellular localisation. Int. J. Cancer 147, 1528–1534. 10.1002/ijc.32900 32010971 PMC7496346

[B23] Liu Q. Yu S. Zhao W. Qin S. Chu Q. Wu K. (2018). EGFR-TKIs resistance via EGFR-independent signaling pathways. Mol. Cancer 17, 53. 10.1186/s12943-018-0793-1 29455669 PMC5817859

[B24] Masanas M. Masia N. Suarez-Cabrera L. Olivan M. Soriano A. Majem B. (2021). The oral KIF11 inhibitor 4SC-205 exhibits antitumor activity and potentiates standard and targeted therapies in primary and metastatic neuroblastoma models. Clin. Transl. Med. 11, e533. 10.1002/ctm2.533 34709738 PMC8516339

[B25] Mcgranahan N. Favero F. De Bruin E. C. Birkbak N. J. Szallasi Z. Swanton C. (2015). Clonal status of actionable driver events and the timing of mutational processes in cancer evolution. Sci. Transl. Med. 7, 283ra54. 10.1126/scitranslmed.aaa1408 PMC4636056 25877892

[B26] Mesaros E. F. Ott G. R. Dorsey B. D. (2014). Anaplastic lymphoma kinase inhibitors as anticancer therapeutics: A patent review. Expert Opin. Ther. Pat. 24, 417–442. 10.1517/13543776.2014.877890 24476492

[B27] Rotow J. Bivona T. G. (2017). Understanding and targeting resistance mechanisms in NSCLC. Nat. Rev. Cancer 17, 637–658. 10.1038/nrc.2017.84 29068003

[B28] Roy B. Han S. J. Y. Fontan A. N. Jema S. Joglekar A. P. (2022). Aurora B phosphorylates Bub1 to promote spindle assembly checkpoint signaling. Curr. Biol. 32, 237–247. 10.1016/j.cub.2021.10.049 34861183 PMC8752509

[B29] Santamaria D. Barriere C. Cerqueira A. Hunt S. Tardy C. Newton K. (2007). Cdk1 is sufficient to drive the mammalian cell cycle. Nature 448, 811–815. 10.1038/nature06046 17700700

[B30] Schafer J. M. Lehmann B. D. Gonzalez-Ericsson P. I. Marshall C. B. Beeler J. S. Redman L. N. (2020). Targeting MYCN-expressing triple-negative breast cancer with BET and MEK inhibitors. Sci. Transl. Med. 12, eaaw8275. 10.1126/scitranslmed.aaw8275 32161105 PMC7427123

[B31] Sequist L. V. Waltman B. A. Dias-Santagata D. Digumarthy S. Turke A. B. Fidias P. (2011). Genotypic and histological evolution of lung cancers acquiring resistance to EGFR inhibitors. Sci. Transl. Med. 3, 75ra26. 10.1126/scitranslmed.3002003 PMC3132801 21430269

[B32] Sung H. Ferlay J. Siegel R. L. Laversanne M. Soerjomataram I. Jemal A. (2021). Global cancer statistics 2020: GLOBOCAN estimates of incidence and mortality worldwide for 36 cancers in 185 countries. Ca. Cancer J. Clin. 71, 209–249. 10.3322/caac.21660 33538338

[B33] Szklarczyk D. Franceschini A. Kuhn M. Simonovic M. Roth A. Minguez P. (2011). The STRING database in 2011: Functional interaction networks of proteins, globally integrated and scored. Nucleic Acids Res. 39, D561–D568. 10.1093/nar/gkq973 21045058 PMC3013807

[B34] Tanaka K. Yu H. A. Yang S. Han S. Selcuklu S. D. Kim K. (2021). Targeting Aurora B kinase prevents and overcomes resistance to EGFR inhibitors in lung cancer by enhancing BIM- and PUMA-mediated apoptosis. Cancer Cell 39, 1245–1261 e6. 10.1016/j.ccell.2021.07.006 34388376 PMC8440494

[B35] Tang Z. Li C. Kang B. Gao G. Li C. Zhang Z. (2017). Gepia: A web server for cancer and normal gene expression profiling and interactive analyses. Nucleic Acids Res. 45, W98–W102. 10.1093/nar/gkx247 28407145 PMC5570223

[B36] Tulchinsky E. Demidov O. Kriajevska M. Barlev N. A. Imyanitov E. (2019). Emt: A mechanism for escape from EGFR-targeted therapy in lung cancer. Biochim. Biophys. Acta. Rev. Cancer 1871, 29–39. 10.1016/j.bbcan.2018.10.003 30419315

[B37] Wang L. Zhang J. Wan L. Zhou X. Wang Z. Wei W. (2015). Targeting Cdc20 as a novel cancer therapeutic strategy. Pharmacol. Ther. 151, 141–151. 10.1016/j.pharmthera.2015.04.002 25850036 PMC4457591

[B38] Wang Z. Wan L. Zhong J. Inuzuka H. Liu P. Sarkar F. H. (2013). Cdc20: A potential novel therapeutic target for cancer treatment. Curr. Pharm. Des. 19, 3210–3214. 10.2174/1381612811319180005 23151139 PMC4014638

[B39] Wei C. Y. Zhu M. X. Lu N. H. Peng R. Yang X. Zhang P. F. (2019). Bioinformatics-based analysis reveals elevated MFSD12 as a key promoter of cell proliferation and a potential therapeutic target in melanoma. Oncogene 38, 1876–1891. 10.1038/s41388-018-0531-6 30385854 PMC6462865

[B40] Wu Y. Lin Y. Pan J. Tu X. Xu Y. Li H. (2021). NCAPG promotes the progression of lung adenocarcinoma via the TGF-beta signaling pathway. Cancer Cell Int. 21, 443. 10.1186/s12935-021-02138-w 34419073 PMC8380402

[B41] Zhou J. Ye J. Zhao X. Li A. Zhou J. (2008). JWA is required for arsenic trioxide induced apoptosis in HeLa and MCF-7 cells via reactive oxygen species and mitochondria linked signal pathway. Toxicol. Appl. Pharmacol. 230, 33–40. 10.1016/j.taap.2008.01.041 18387645

