# Supplementary figures and images for: Identification and verification of hub genes associated with the progression of non-small cell lung cancer by integrated analysis
Source: Front Pharmacol. 2022 Sep 13;13:997842. doi: 10.3389/fphar.2022.997842 (PMC9513139; doi:10.3389/fphar.2022.997842)

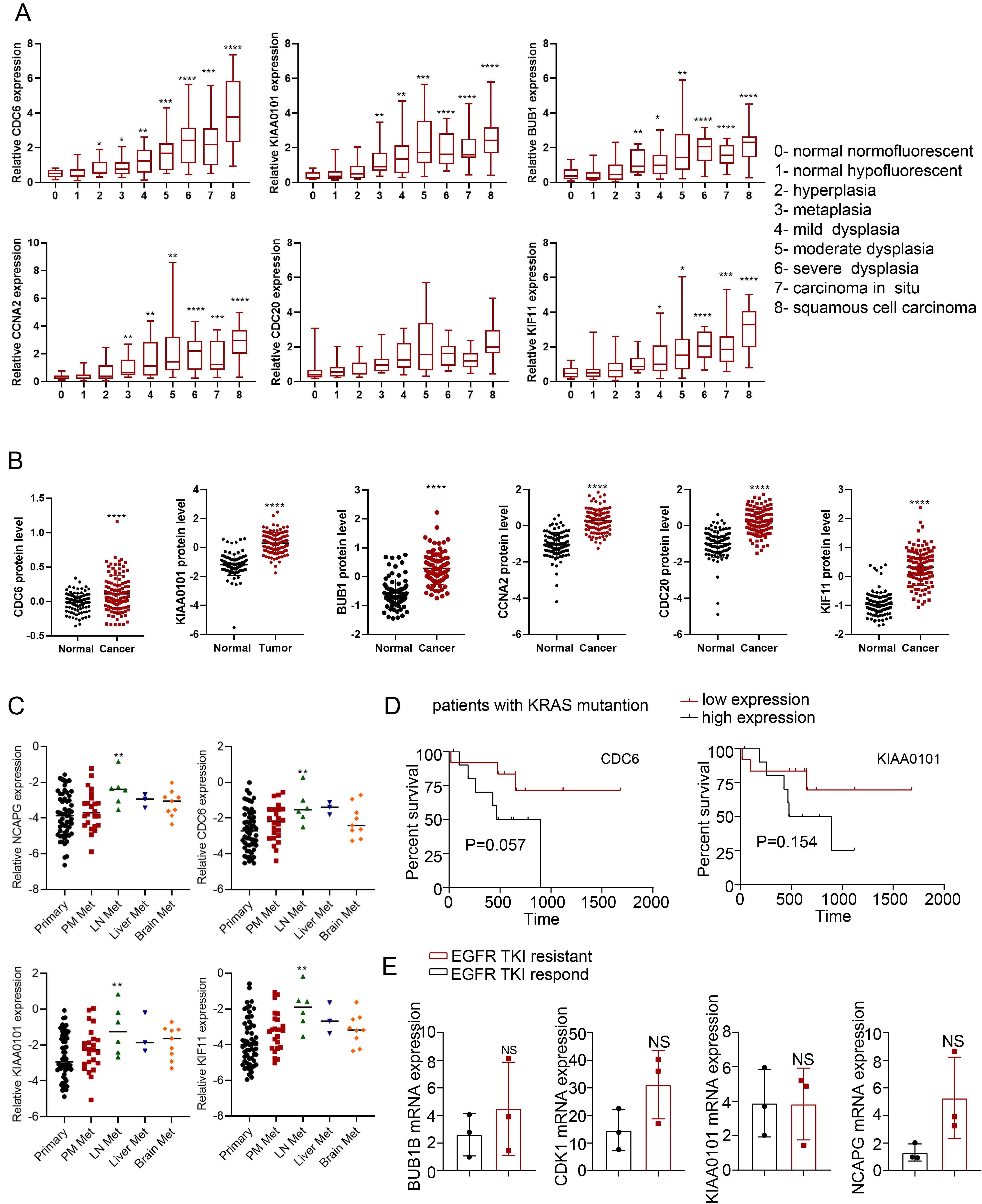

Supplement: Supplementary file 2 [file Image3.JPEG]

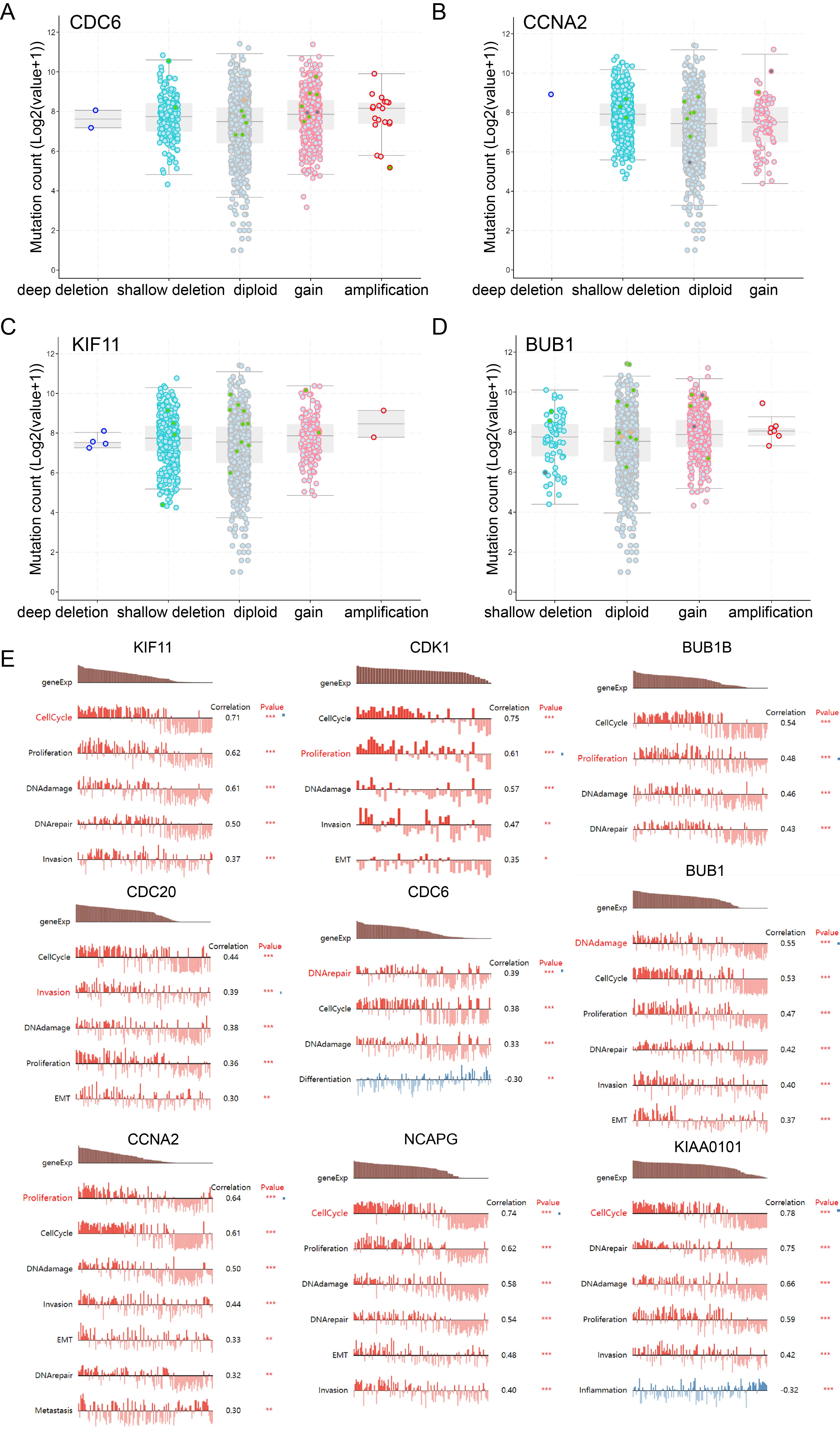

Supplement: Supplementary file 5 [file Image4.JPEG]

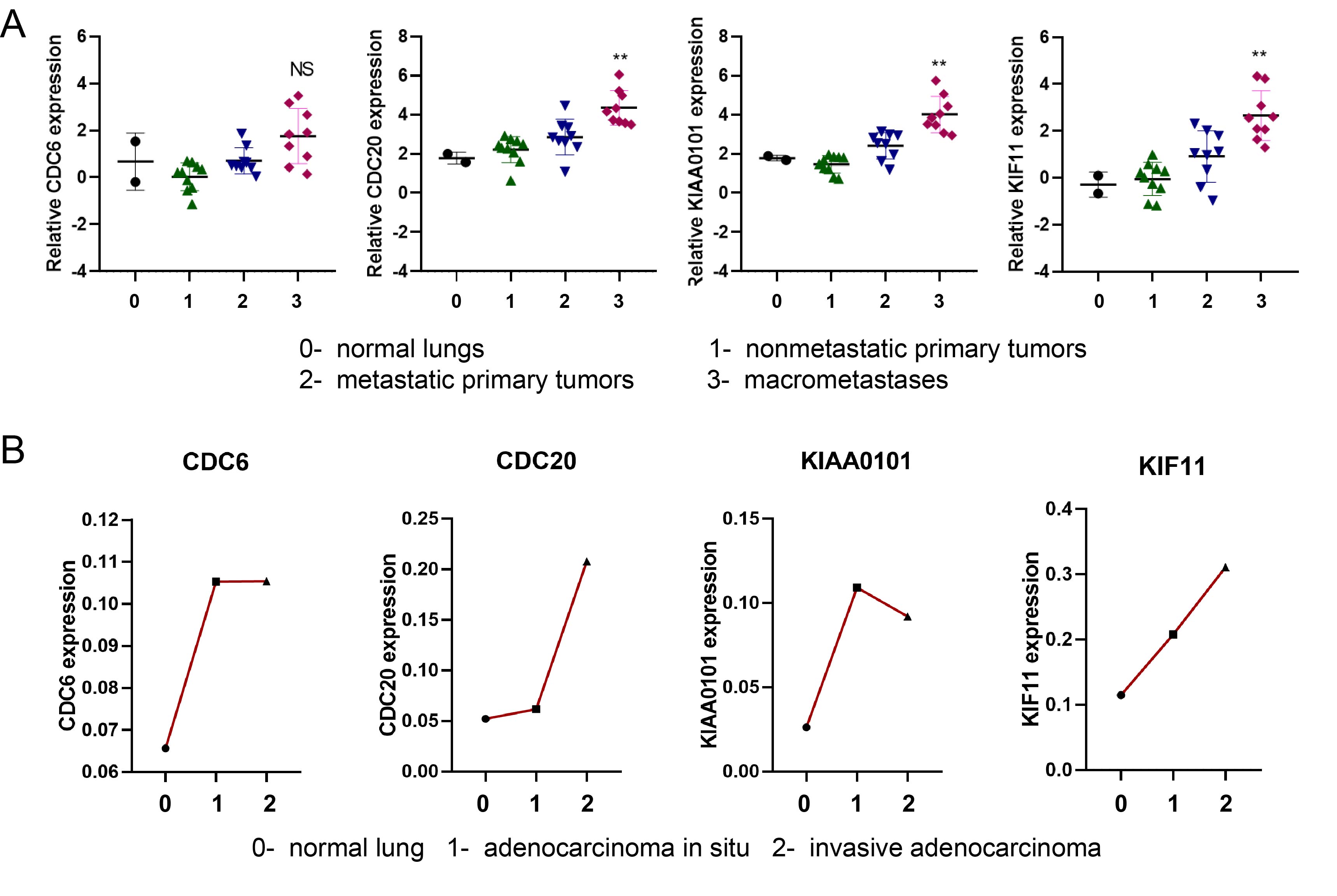

Supplement: Supplementary file 7 [file Image2.JPEG]

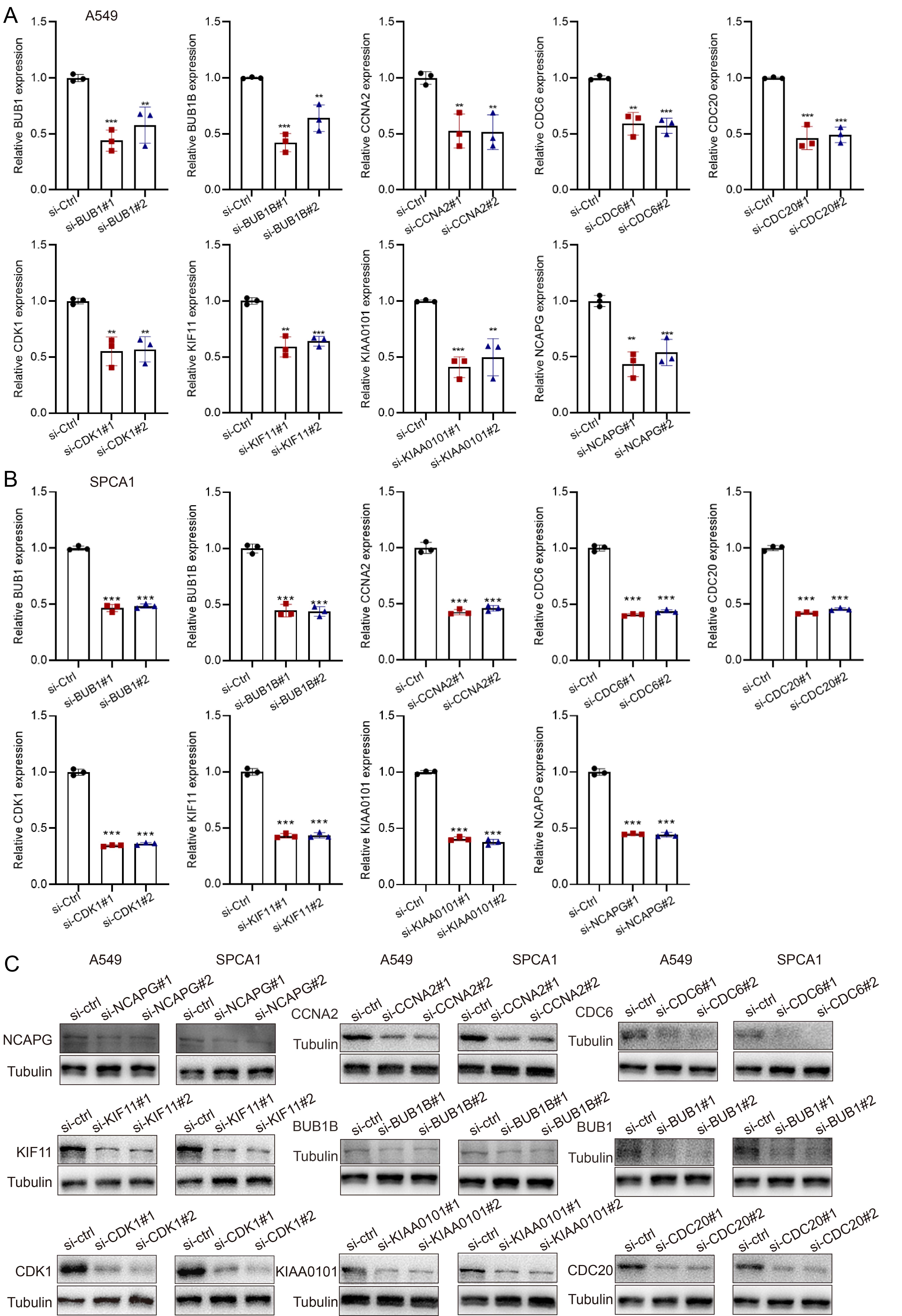

Supplement: Supplementary file 8 [file Image5.JPEG]

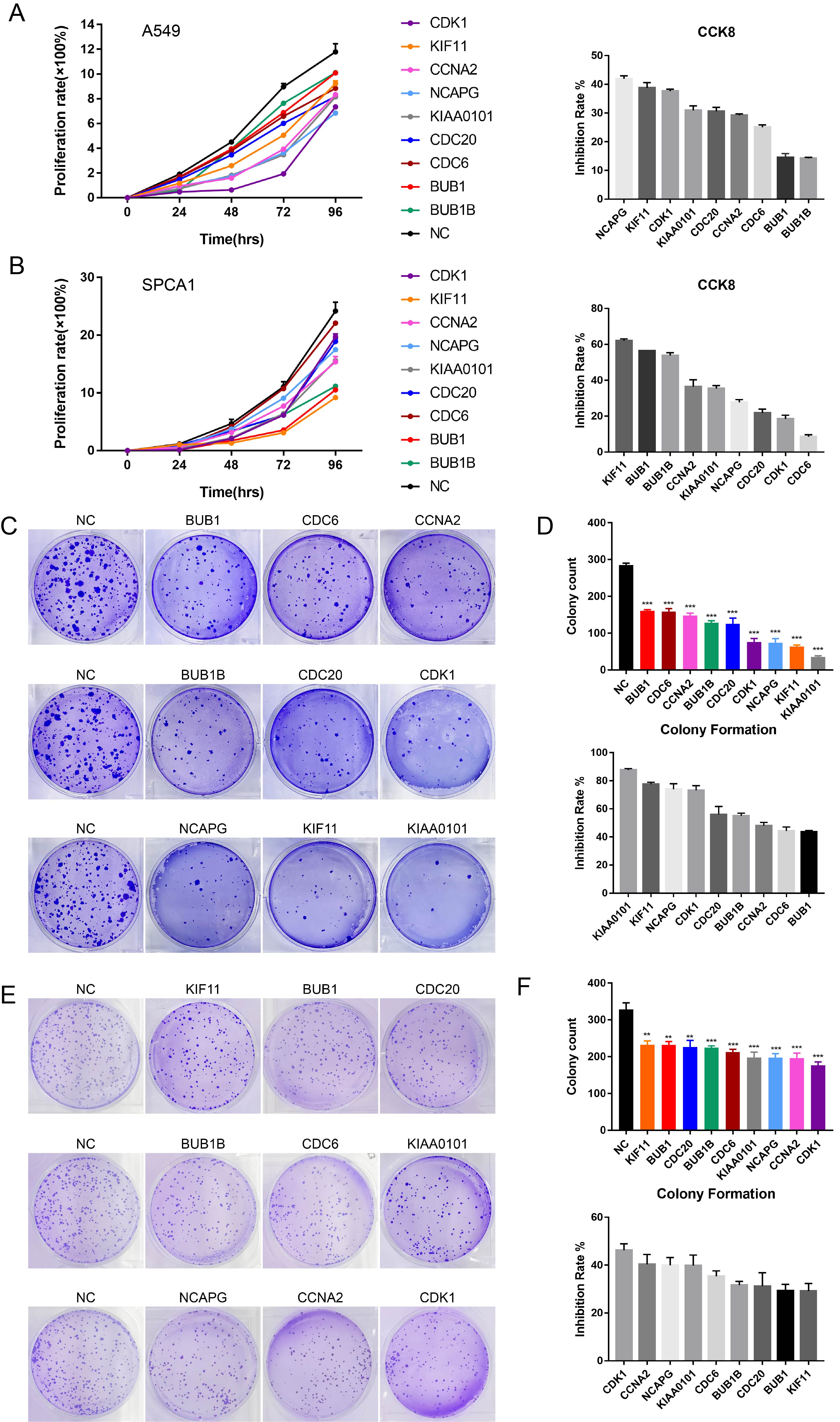

Supplement: Supplementary file 12 [file Image6.JPEG]
